# ‘Arm‐based’ parameterization for network meta‐analysis

**DOI:** 10.1002/jrsm.1187

**Published:** 2015-11-27

**Authors:** Neil Hawkins, David A. Scott, Beth Woods

**Affiliations:** ^1^ ICON Health EconomicsOxfordOX2 0JJUK; ^2^ Centre for Health Economics, Alcuin ‘A’ BlockUniversity of YorkYorkYO10 5DDUK; ^3^ Present address: Department of Health Services Research and PolicyLondon School of Hygiene and Tropical MedicineLondonUK

**Keywords:** network meta‐analysis, arm‐based parameterization, winBUGS

## Abstract

We present an alternative to the contrast‐based parameterization used in a number of publications for network meta‐analysis. This alternative “arm‐based” parameterization offers a number of advantages: it allows for a “long” normalized data structure that remains constant regardless of the number of comparators; it can be used to directly incorporate individual patient data into the analysis; the incorporation of multi‐arm trials is straightforward and avoids the need to generate a multivariate distribution describing treatment effects; there is a direct mapping between the parameterization and the analysis script in languages such as WinBUGS and finally, the arm‐based parameterization allows simple extension to treatment‐specific random treatment effect variances.

We validated the parameterization using a published smoking cessation dataset. Network meta‐analysis using arm‐ and contrast‐based parameterizations produced comparable results (with means and standard deviations being within +/− 0.01) for both fixed and random effects models. We recommend that analysts consider using arm‐based parameterization when carrying out network meta‐analyses. © 2015 The Authors *Research Synthesis Methods* Published by John Wiley & Sons Ltd.

## 1 Introduction

Network meta‐analysis combines evidence from trials comparing different sets of treatments in a single coherent analysis (Lu and Ades, 2004; Caldwell *et al.,* 2005; Jansen *et al.,* 2011) providing estimates of relative treatment effects that are informed by both direct and indirect evidence. Methods for network meta‐analysis have been studied and extended considerably in recent years.

In this paper, we consider the parameterization of network meta‐analyses. The parameterization of an analysis is the structure that links model parameters to data. Alternative parameterizations can give the same results, but may differ in their complexity and the difficulty with which specific analytic features can be incorporated. This paper describes an alternative parameterization for network meta‐analysis to that given in the National Institute for Health and Care Excellence Decision Support Unit Technical Support Document 2, TSD2 (Dias *et al.,* 2014) and a number of other publications (e.g. Cooper *et al.,* 2009). In keeping with these documents, we implement the arm‐based parameterization within a Bayesian framework.

The parameterization given in TSD2 directly models average treatment effects; hence we refer to this as a ‘contrast‐based’ parameterization. Our alternative parameterization directly models the responses observed in individual treatment arms; hence we refer to this as an ‘arm‐based’ parameterization. It is worth noting that in trials we do not directly observe average treatments effects, such as risk differences or odds ratios; rather we observe responses in individual patients allocated to different trial arms, from which we derive these statistics.

The choice of contrast‐based and arm‐based parameterizations is distinct from the structure of the available data which may take the form of contrast‐ or arm‐level statistics (which are discussed in TSD2 and Salanti *et al.,* 2008). Arm‐level statistics describe the outcomes in individual trial arms (e.g. how many events were observed in a certain number of patients), whereas contrast‐level statistics describe differences between trial arms (e.g. using an odds ratio). We demonstrate that both contrast‐ and arm‐based *parameterizations* can be used to model contrast‐ and arm‐level *statistics*. The main body of this paper describes the modelling of arm‐level statistics, and the extension to contrast‐level statistics is described in the online supporting information.

The arm‐based parameterization differs from the contrast‐based parameterization in three important ways. First, the arm‐based parameterization directly models the observed responses in individual trial arms. This simplifies the model code and allows for the use of a normalized (“long and narrow”) data format with one row per trial *arm*, as opposed to a non‐normalized (“short and wide”) data format with one row per *trial*. A normalized data format has a constant structure regardless of the number of arms in the included trials. This feature is particularly beneficial for individual patient data analyses where the data may include one row per patient.

Second, the arm‐based parameterization treats random treatment effects as a variation in responses at the arm level. This has two advantages: it avoids the need to explicitly model the correlation in random treatment effect variation in studies with more than two arms (e.g. by simulating a multivariate normal distribution), and it requires only a simple modification to the analysis code to allow random effects variances to vary between treatment contrasts.

Finally, when using arm‐based parameterization, the variation of the estimated value of the trial‐level response term can be used directly as a measure of heterogeneity in reference treatment risk (which may reflect differences in trial design, prognostic factors or random variation).

Although it has been suggested in the literature that the arm‐based parameterization is ‘not identified’ (see the Appendix of Dias *et al.,* 2012), we argue in this paper that it is (see Discussion), and we also demonstrate empirically that this model produces equivalent results to the contrast‐based model. This result is tested for network structures in which some treatments are not directly compared to the reference treatment in order to demonstrate that the result is generalizable to other network structures. We provide example WinBUGS code to encourage readers to explore the application of arm‐based parameterization.

## 2 Methods

### 2.1 Overview

The implementation of the arm‐based parameterization is illustrated in an analysis of the smoking cessation data set referred to in the TSD4 (Dias *et al.,* 2013a). This dataset has been used in a number of previous methodological studies (Hasselblad, 1998; Ades *et al.,* 2006; Lu and Ades, 2006; Dias *et al.,* 2010). The results of a network meta‐analysis using the arm‐based parameterization are compared with the results obtained using the contrast‐based parameterization for both fixed and random treatment effects models. Results are also shown for a random effects model in which the random effect variance is allowed to vary by treatment.

### 2.2 Example dataset

**Figure 1:**
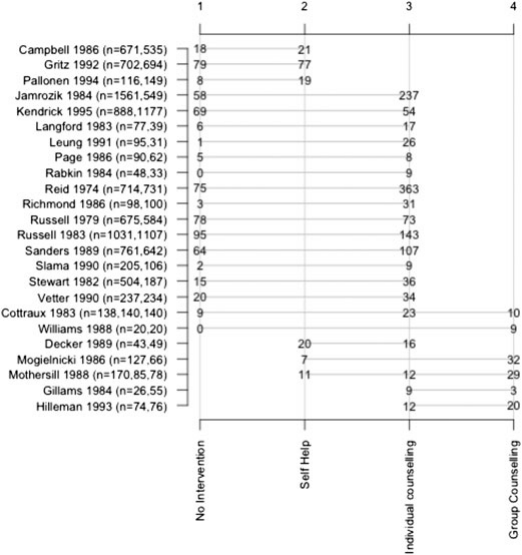
Network of evidence for the trials of smoking‐cessation interventions. A line joins the interventions compared in each trial; the number on an intervention indicates the number of individuals with successful smoking cessation at 6−12 months.

The original dataset included 24 randomized controlled trials comparing four alternative smoking cessation treatments: no contact; self‐help; individual counselling and group counselling. The resulting network is shown in Figure 1. The endpoint is the number of individuals with successful smoking cessation at 6–12 months. Data for this analysis were extracted from Hasselblad (1998). The Cottraux *et al.* (1983) and Williams and Hall (1988) studies were removed in this analysis to produce a network including one treatment (group counselling) that was not directly compared with the reference treatment (no intervention). In the reduced dataset, the only paths between these two treatments are indirect. This change demonstrates that the modified parameterization could be applied to networks with this structure (Salanti *et al.,* 2008). The dataset also includes a three‐arm trial. An analysis of the full dataset is also conducted to provide further validation.

The following sections describe fixed effect and random effects models using the contrast‐based parameterization (Contrast‐FE and Contrast‐RE) and the arm‐based parameterization (Arm‐FE and Arm‐RE), and finally the arm‐based parameterization with treatment‐specific random effect variances (Arm‐RE Tx). Code for these models is provided in the online Supporting Information.

### 2.3 Parameterizations for network meta‐analysis

We use the following notation to describe the network meta‐analysis parameterizations: there are *K* treatments labelled *k = 1,2,…,K,* and *J* trials labelled *j = 1,2,…,J*. Treatment *1* is the overall reference treatment for the network meta‐analysis. In the *j^th^* trial the number of subjects experiencing events in treatment arm *k* is *r_jk_* out of a total of *n_jk_* subjects. A binomial distribution is assumed for these data:

(1) $$r_{jk}\sim \;\text{Bin}\left(p_{jk}n_{jk}\right)$$

where *p_jk_* is the probability of an event in trial *j* under treatment *k*. The probability *p_jk_* is derived from the log odds of an event (*η*_*jk*_):

(2) $$p_{jk}=\frac{exp\left(\eta_{jk}\right)}{1+exp\left(\eta_{jk}\right)}\text{.}$$

#### 2.3.1 Contrast‐based parameterization—fixed effects (Contrast‐FE)

In the contrast‐based parameterization for a fixed effects network meta‐analysis, the log odds *η*_*jk*_ are assumed to be generated by the following model:

(3) $$\eta_{jk}=\;\left\{\begin{array}{c} \mu_{j}\;;\;\text{if}\;k=b_{j}\\ \mu_{j}+\;d_{b_{j}k}\;;\;\text{if}\;k>b_{j} \end{array}\right.\text{.}$$

μ_j_ is the log odds of an event in trial *j* on the base treatment for that trial, *b_j_*. Each treatment in the analysis is assigned a number, and the base treatment is (arbitrarily) defined as the lowest numbered treatment within each trial. $d_{b_{j}k}$ is a functional parameter and describes the log odds ratio comparing treatment *k* to treatment *b_j_*. Note that some authors reverse the subscripts (e.g. Salanti *et al.,* 2008). A set of consistency equations relate the functional parameters to the basic parameters: $d_{b_{j}k}\;=d_{1k}\;-d_{1b_{j}}$, where *d*_11_ = 0. The inclusion of μ_j_ ensures that between trial differences in absolute response do not affect estimates of treatment effect. This is sometimes referred to as ‘respecting the trials' randomization’ in the literature.

#### 2.3.2 Contrast‐based parameterization—random effects (Contrast‐RE)

In the random treatment effects analysis, trial‐specific treatment effects are assumed to vary around the overall mean treatment effect.

(4) $$\eta_{jk}=\;\left\{\begin{array}{c} \mu_{j}\;;\;\text{if}\;k=b_{j}\\ \mu_{j}+\;\delta_{jb_{j}k};\;\text{if}\;k>b_{j} \end{array}\right.$$

For networks including only two arm trials, the random effects ( $\delta_{jb_{j}k}$) are assumed to follow a normal distribution. The random effect variance is denoted *σ* ^2^ and represents the between‐trial variability in treatment effects:

(5) $$\delta_{jb_{j}k}\sim N\left(d_{b_{j}k,}\sigma^{2}\right)\text{.}$$

For networks including multi‐arm trials (trials with three or more arms), the random effects for the various contrasts should be correlated, as the treatment effects within a trial are constrained to be consistent. For a random effects analysis where *σ* ^2^ is assumed constant across the treatment contrasts, the random effects are typically assumed to follow a multivariate normal distribution as shown in equation 6 (Dias *et al.,* 2014).

(6) $$\delta_{j}=\left(\begin{array}{c} \delta_{jb_{j}k_{1}}\\ \vdots \\ \delta_{jb_{j}k_{L}} \end{array}\right)\sim N\left(\left[\begin{array}{c} d_{b_{j}k_{1}}\\ \vdots \\ d_{b_{j}k_{L}} \end{array}\right],\left[\begin{array}{cccc} \sigma^{2} & \frac{\sigma^{2}}{2} & \ldots \; & \frac{\sigma^{2}}{2}\\ \frac{\sigma^{2}}{2} & \sigma^{2} & \ldots & \frac{\sigma^{2}}{2}\\ \vdots & \vdots & \ddots & \vdots \\ \frac{\sigma^{2}}{2} & \frac{\sigma^{2}}{2} & \ldots & \sigma^{2} \end{array}\right]\right)$$

δ_*j*_ is the vector of random treatment effects for trial *j*. Trial *j* includes *L* + 1 treatments *b_j_, k_1_, k_2_, …, k_L_.* The vector *δ*_*j*_ therefore includes *L* comparisons to the trial‐specific base treatment *b_j_*. The multivariate normal distribution is generated using a series of conditional univariate normal distributions for the random effects of arms indexed *l* > 1 (Dias *et al.,* 2014). As in the fixed effects model, a set of consistent log odds ratios $d_{b_{j}k}$ are identified by expressing each as a function of the basic parameters: $d_{b_{j}k}\;=d_{1k}\;-d_{1b_{j}}$, where *d*_11_ = 0. The constant variance and multivariate normality assumptions may not be valid. These assumptions are rarely examined as the available data are not usually rich enough to explore alternative assumptions.

#### 2.3.3 Arm‐based parameterization—fixed effects (Arm‐FE)

In arm‐based parameterization, individual trial treatment arms are denoted by *i* = 1, 2, …, *I* where *I* is the total number of arms in the *J* trials. The subscript *jk* in equations 1 and 2 can therefore be replaced by the single subscript *i*. *s*[*i*] denotes the trial to which arm *i* belongs and *t*[*i*] denotes the treatment received by patients in this arm. The treatment effect for treatment *t* compared with the reference treatment (*t = 1*) is denoted by *d*_*t*_. *d*_1_ is constrained to 0. This notation corresponds directly to the code and allows the data to be arranged with one row per arm *i*.

The log odds for arm *i* (*η*_*i*_) are assumed to be generated by the following model:

(7) $$\eta_{i}=\mu_{s\left[i\right]}+d_{t\left[i\right]}\text{.}$$

*μ*_*s*[*i*]_ is a trial‐level fixed effect term representing the response to the reference treatment. The variation in *μ*_*s*[*i*]_ across studies provides a measure of the heterogeneity in reference treatment risk across studies and can provide some indication of whether heterogeneity and/or inconsistency are likely to be present (Achana *et al.,* 2013). As in contrast‐based parameterization, this term is included to ensure that between trial differences in absolute response do not affect estimates of treatment effect. In this model, the values for the *μ*_*s*[*i*]_ parameters are directly comparable across trials (regardless of trial comparators) as they correspond to the predicted log odds of response to the reference treatment (the treatment with index 1). The *μ*_*s*[*i*]_ represent the trial‐specific outcome for the reference treatment, regardless of whether this treatment is included in the trial, and are generated under the assumption that the consistency model is correct.

#### 2.3.4 Base‐treatment shift parameterization

In this variant of the Arm‐FE model, equation 7 is exchanged with

(8) $$\eta_{i}=\mu {'}_{s\left[i\right]}+d_{t\left[i\right]}-d_{b\left[i\right]}$$

where *b*[*i*] is (arbitrarily) the lowest treatment index for trial *s*[*i*] and *μ* ' _*s*[*i*]_ represents the predicted log odds of the base treatment in trial *s*[*i*] (i.e. *μ* ' _*s*[*i*]_ = *μ*_*s*[*i*]_ + *d*_*b*[*i*]_) (Woods *et al.,* 2010). We refer to this as the ‘Base‐treatment shift’ model as the additional term (−*d*_*b*[*i*]_) shifts the trial‐level base treatment term (*μ* ' _*s*[*i*]_) to represent outcomes in the trial‐specific base treatment arm, *b*[*i*]. This model may lead to faster convergence in some cases. Note, in this model the values for the *μ* ' _*s*[*i*]_ parameters are not directly comparable across all trials as they correspond to the response to the lowest indexed treatment *within* each trial.

#### 2.3.5 Arm‐based parameterization—random treatment effects with common variance (Arm‐RE)

In the arm‐based parameterization, the multivariate normal distribution is generated by replacing the fixed treatment effect in equation 7 with a trial arm‐specific random effect (*δ*_*s*[*i*],*t*[*i*]_).

(9) $$\eta_{i}=\mu_{s\left[i\right]}+\delta_{s\left[i\right],t\left[i\right]}$$

where

(10) $$\delta_{s\left[i\right],t\left[i\right]}\sim N\left(d_{t\left[i\right]},\frac{\sigma^{2}}{2}\right)\text{.}$$

The trial arm‐specific random effects (*δ*_*s*[*i*],*t*[*i*]_) are assumed to be uncorrelated within and across trials. As in the contrast‐based parameterization, *σ* ^2^ represents the random effect variance. As this is assumed to be constant across all contrasts within the network, each individual treatment response is associated with a random effect variance of $\frac{\sigma^{2}}{2}$. This reflects the variation in response observed in individual arms.

The correlation in the random variation in treatment effects within multi‐arm trials arises as treatment effect estimates within each trial are jointly dependent on the variation in response in the base treatment arm of the trial. The model in equations 9 and 10 produces the correct covariance for the common variance random effects model. For example, for a single trial *s* comparing treatments 1, 2 and 3, the random effect variance for the treatment effect contrast comparing treatments 1 and 2 is

(11) $$var\left(\delta_{s,2}-\delta_{s,1}\right)=\frac{\sigma^{2}}{2}+\frac{\sigma^{2}}{2}=\sigma^{2}\text{.}$$

The covariance of the random effects for the contrasts comparing treatments 1 and 2 and treatments 1 and 3 is

(12) $$cov\left(\delta_{s,2}-\delta_{s,1},\delta_{s,3}-\delta_{s,1}\right)=cov\left(\delta_{s,1},\delta_{s,1}\right)=\frac{\sigma^{2}}{2}\text{.}$$

Arm‐based parameterization therefore avoids the need to use a multivariate normal distribution to reflect this covariance.

In both contrast‐ and arm‐based parameterization, the random effect variance provides a measure of the variation in observed treatment effects (beyond the variation attributable to random variation within trials) from the predictions of the consistency model. The random effect variance therefore captures both heterogeneity in treatment effects within pair‐wise comparisons and inconsistency between direct and indirect estimates of treatment effects. Inconsistency occurs because of the possibility of conflicts between estimates of treatment effects from trials directly comparing the treatments (e.g. A vs. B) and indirect estimates of the treatment effects (e.g. from A vs. C and B vs. C trials). Inconsistency arises when effect modifiers are present and the distribution of effect modifiers differs between the direct and indirect evidence (Dias *et al.,* 2013a). The random effect variance captures any deviations from the underlying model of single and consistent treatment effects and will therefore reflect both heterogeneity and inconsistency.

#### 2.3.6 Random treatment effects with treatment‐specific variance (Arm‐RE Tx)

Network meta‐analysis models typically assume that random effect variances are equal for all contrasts. However, this assumption may not hold if, for example, one of the interventions in the network is likely to be associated with more variable outcomes. (Lu and Ades (2009) give the example of a surgical intervention in a network of drug treatments.) Attempts to relax this assumption in the applied literature have been limited both by the availability of sufficient data to inform models including multiple random effect parameters and the complexity of the available methods which focus on allowing differences in random effect variance at the contrast level (Lu and Ades, 2009; Salanti, 2012).

By assuming that differences in random effect variance occur at the level of the individual treatment, rather than at the level of treatment contrasts, we can readily adapt the Arm‐RE model to allow the random effect variance to vary by treatment:

(13) $$\delta_{s\left[i\right],t\left[i\right]}\sim N\left(d_{t\left[i\right]},\frac{\sigma_{t\left[i\right]}^{2}}{2}\right)\text{.}$$

### 2.4 Model estimation

Parameter values were estimated using Markov chain Monte Carlo techniques as implemented in WinBUGS. Three chains were run starting from different initial values. The same set of vague priors (*μ*_*j*_ ~ *N*(0, 10^4^), *μ*_*s*[*i*]_ ~ *N*(0, 10^4^); *d*_1*k*_ ~ *N*(0, 10^4^), *σ* ~ *Unif*(0, 5)) and three sets of initial values were used across all models (see online Supporting Information for further detail). Models were run for 20 000 iterations as a burn‐in period and a further 20 000 iterations are used for estimation. Convergence was assessed using Brooks Gelman Rubin plots (Brooks and Gelman, 1998) and by examining trace plots; no thinning was required. Adequacy of Monte Carlo sampling error was judged using the Rhat statistic (Brooks and Gelman, 1998). Model fit was compared using the deviance information criterion (DIC) (Spiegelhalter *et al.,* 2002).

## 3 Results

The results from the analyses incorporating fixed treatment effects are presented in Table 1. The contrast‐based (Contrast‐FE) and arm‐based (Arm‐FE) parameterizations produced similar results with differences in estimated parameter means and standard deviations of less than ±0.01. The deviance and DIC measures of model fit are also similar. The fixed effects analysis with base‐treatment shift parameterization also produced similar results (within ±0.01).

**Table 1:** Fixed treatment effects.

Model results	Treatment	Contrast‐based fixed treatment effects (Contrast‐FE)		Arm‐based fixed treatment effects (Arm‐FE)		Arm FE‐base treatment shift parameterization	
		Mean	SD	Mean	SD	Mean	SD
Treatment effects (log odds ratios vs. no intervention)	*Self‐help*	0.25	0.13	0.25	0.13	0.25	0.13
	*Individual counselling*	0.75	0.06	0.75	0.06	0.75	0.06
	*Group counselling*	1.02	0.21	1.02	0.21	1.01	0.21
Deviance		460.0	7.2	460.0	7.1	460.0	7.2
DIC		485.0		485.0		485.0	

FE = fixed effects; RE = random effects; SD = standard deviation; DIC = deviance information criterion.

Table 2 presents the results for random treatment effects with common variance. Again, the contrast‐based (Contrast‐RE) and arm‐based (Arm‐RE) parameterizations produced similar results (within ±0.01).

**Table 2:** Random treatment effects.

Model results	Treatment	Contrast‐based random treatment effects (Contrast‐RE)		Arm‐based random treatment effects (Arm‐RE)		Arm‐based random treatment effects, non‐constant variance (Arm‐RE Tx)	
		Mean	SD	Mean	SD	Mean	SD
Treatment effects (log odds ratios vs. no intervention)	*Self‐help*	0.46	0.39	0.46	0.40	0.47	0.45
	*Individual counselling*	0.77	0.24	0.78	0.23	0.81	0.28
	*Group counselling*	1.09	0.51	1.09	0.51	1.07	0.66
Random effect SD	Common	0.79	0.18	0.79	0.18	—	—
	*No contact*					0.92	0.49
	*Self‐help*					0.65	0.61
	*Individual counselling*					0.80	0.49
	*Group counselling*					1.05	0.97
Deviance		258.0	9.4	258.1	9.4	257.2	9.4
DIC		298.6		298.6		298.4	

FE = fixed effects; RE = random effects; SD = standard deviation; DIC = deviance information criterion.

Finally, the results from the analyses incorporating treatment‐specific random effect variances (Arm‐RE Tx) are shown in the final columns of Table 2. The treatment‐specific random effects SDs (means ranging from 0.65 to 1.05) are similar in magnitude to the common random effect SD (mean 0.79). Models that allow the random effect variance to vary by treatment comparison require additional parameters; in this case three are required. This is reflected in the increased uncertainty around the random effect estimates. However, the DICs for the model with a single random effect variance and the model with treatment‐specific variances are similar at 298.6 and 298.4, respectively.

Arm‐based parameterization models produced comparable results to the published contrast‐based models for the full dataset. These results are presented in the online Supporting Information.

## 4 Discussion

The arm‐based parameterization for network meta‐analysis produced comparable results to the contrast‐based parameterization more commonly used in the literature. This is to be expected given the equivalence of the parameterizations. The arm‐based parameterization offers four main advantages.

### (i) Simplified data formatting

It allows the data to be structured with a row for each trial arm, a ‘long and narrow’ format, rather than the ‘wide’ format where each row represents a study required by contrast‐based parameterization. The normalized ‘long and narrow’ format is easier to manipulate and therefore less likely to be subject to data manipulation errors.

The ‘long and narrow’ data format in particular facilitates individual patient data network meta‐analysis, as each patient's data will usually occupy a separate row. Incorporating this type of data within the contrast‐based parameterization therefore requires significant modifications to the data and/or code. These are not necessary when the arm‐based code is used.

### (ii) Simplified random effects modelling for multi‐arm trials

The arm‐based parameterization splits the random effect into arm‐level components. The analysis code does not require modification to incorporate the within trial correlation in random effects on treatment contrasts for multi‐arm trials. This avoids the considerable complexity involved in simulating a multivariate distribution of random effects on treatment contrasts (using conditional univariate distributions) required by the contrast‐based parameterization.

### (iii) Modelling treatment‐specific random effect variances

This approach to modelling random treatment effects also allows a simple extension to treatment‐specific random effect variances. The resulting model has a simple interpretation as representing differences in the variance in response to specific treatments. These differences are particularly important if the predictive distribution of the treatment effects is used to represent our beliefs about future efficacy (Lu and Ades, 2009). The assumption that differences in random effects occur at the arm level is plausible in many cases. For example, the responses in usual care arms may be more variable than active treatment arms, or some interventions may be subject to individual titration or involve significant clinical expertise (e.g. surgery, manual physiotherapy). Existing models (see Lu and Ades, 2009) assume that differences in variability occur at the level of the treatment contrast. This may be appropriate in some circumstances, for example, if all comparisons of generic drugs were conducted in pragmatic trials, but all comparisons of new drugs to generic drugs were conducted as registration trials. We believe that assuming that the random effect variance differs by treatment rather than treatment contrast is likely to be the more realistic assumption for many applied studies.

### (iv) Direct estimate of reference treatment outcomes

The *μ*_*s*[*i*]_ terms generated by arm‐based parameterization can be compared across trials as they all represent the predicted response to the reference treatment. In contrast‐based parameterization, the *μ*_*j*_ terms represent the response associated with the (arbitrary) base treatment in each trial. In contrast‐based parameterization, the predicted response to the reference treatment therefore needs to be derived (as $\mu_{j}-d_{1b_{j}}$). Using arm‐based parameterization simplifies the estimation of this quantity. This may be particularly useful when including reference treatment risk as a treatment‐effect modifier in meta‐regression (Achana *et al.,* 2013). Use of reference treatment risk as both a parameter and treatment‐effect modifier is an established approach and allows the uncertainty in reference treatment risk (including its correlation with the treatment effect parameters) to be appropriately reflected in the analysis (Achana *et al.,* 2013; Dias *et al.,* 2013b).

Concerns have been raised about the identification of arm‐based models and their potential to produce unstable parameter estimates (see the Appendix of Dias *et al.,* 2012). However, consider an analysis of two trials, one comparing treatment A vs. B and the other comparing treatment B vs. C, using the fixed treatment effects arm‐based parameterization. There will be four parameters to estimate, two treatment effect parameters and two trial‐specific base treatment effects, and four data points. The model is therefore identified. The number of parameters to be estimated and number of data points remains the same across the models considered in this manuscript. Furthermore, our empirical comparison of the two parameterizations shows that arm‐based parameterization is not unstable and produces equivalent results to contrast‐based parameterization.

Network meta‐analyses methods have been extended to incorporate different types of data and treatment effect scales, to allow analysis of potential inconsistency between direct and indirect data, and to adjust for heterogeneity using meta‐regression. The online Supporting Information demonstrates how the arm‐based parameterization approach to network meta‐analysis can incorporate these extensions.

## 5 Conflict of interest

None of the authors have any conflicts of interest with respect to this work. No funding was received for undertaking this work or preparing the manuscript.

## Supporting information

Supporting info item
