# Supplementary material for: ‘Arm‐based’ parameterization for network meta‐analysis
Source: Res Synth Methods. 2015 Nov 27;7(3):306–13. doi: 10.1002/jrsm.1187 (PMC5063191; doi:10.1002/jrsm.1187)
Supplement: Supplementary file 1 — Supporting info item [file JRSM-7-306-s001.docx]

**Supporting information**

***Additional analysis - full smoking cessation dataset***

*Note: results presented on log scale to match original presentation in Lu and Ades 2006.*

Table 1a: Fixed treatment effects (results as log odds ratios)

| Model Results | Treatment | Contrast-based fixed treatment effects  (Contrast-FE) | | Arm-based fixed treatment effects  (Arm-FE) | |
| --- | --- | --- | --- | --- | --- |
|  |  | mean | Sd | mean | sd |
| Treatment effects  (log odds ratios vs. no intervention) | *Self-help* | 0.22 | 0.13 | 0.23 | 0.13 |
|  | *Individual counselling* | 0.77 | 0.06 | 0.77 | 0.06 |
|  | *Group counselling* | 0.84 | 0.18 | 0.84 | 0.18 |
| Deviance |  | 494.8* | | 494.7 | |
| DIC |  | 521.8* | | 521.8 | |
| Source |  | Lu & Ades (2006) | | Authors’ analysis | |

FE=fixed effects; RE=random effects; sd=standard deviation; DIC=deviance information criterion; . *DIC and Deviance were estimated by re-running the TSD2 code, the values returned by R2Winbugs differed from those reported by Lu & Ades (2006). The treatment effects are as reported by Lu and Ades (2006) and match those returned when the TSD2 code was re-run.

Table 2a: Random treatment effects

| Model Results | Treatment | Contrast-based Random treatment effects  (Contrast-RE) | | Arm-based Random treatment effects  (Arm-RE) | |
| --- | --- | --- | --- | --- | --- |
|  |  | mean | sd | mean | sd |
| Treatment effects  (log odds ratios vs. no intervention) | *Self-help* | 0.49 | 0.40 | 0.49 | 0.40 |
|  | *Individual counselling* | 0.84 | 0.24 | 0.84 | 0.24 |
|  | *Group counselling* | 1.10 | 0.44 | 1.10 | 0.43 |
| Random Effects SD | Common | 0.85* | NA | 0.84 | 0.19 |
| Deviance |  | 281.8** | | 281.7 | |
| DIC |  | 326.8** | | 326.6 | |
| Source |  | Lu & Ades (2006) | | Authors’ analysis | |

FE=fixed effects; RE=random effects; sd=standard deviation; DIC=deviance information criterion; NR = not reported; * = calculated from reported random effect variance of 0.731; **DIC and Deviance were estimated by re-running the TSD2 code, the values returned by R2Winbugs differed from those reported by Lu & Ades (2006). The treatment effects are as reported by Lu and Ades (2006) and match those returned when the TSD2 code was re-run.

***WinBUGS model code***

**Contrast-FE – Contrast-based parameterisation - Fixed Effects (from Dias et al., 2014)**

Note that in this code the studies are index using *i* rather than *j* as in the manuscript text.

# Binomial likelihood, logit link

# Fixed effects model

model{ # *** PROGRAM STARTS

for(i in 1:ns){ # LOOP THROUGH STUDIES

mu[i] ~ dnorm(0,.0001) # vague priors for all trial baselines

for (k in 1:na[i]) { # LOOP THROUGH ARMS

r[i,k] ~ dbin(p[i,k],n[i,k]) # binomial likelihood

# model for linear predictor

logit(p[i,k]) <- mu[i] + d[t[i,k]] - d[t[i,1]]

# expected value of the numerators

rhat[i,k] <- p[i,k] * n[i,k]

#Deviance contribution

dev[i,k] <- 2 * (r[i,k] * (log(r[i,k])-log(rhat[i,k]))

+ (n[i,k]-r[i,k]) * (log(n[i,k]-r[i,k]) - log(n[i,k]-rhat[i,k])))

}

# summed residual deviance contribution for this trial

resdev[i] <- sum(dev[i,1:na[i]])

}

totresdev <- sum(resdev[]) # Total Residual Deviance

d[1]<-0 # treatment effect is zero for reference treatment

# vague priors for treatment effects

for (k in 2:nt){ d[k] ~ dnorm(0,.0001) }

}

# *** PROGRAM ENDS

# Data

# nt=no. treatments, ns=no. studies

list(nt=4,ns=22)

r[,1] n[,1] r[,2] n[,2] r[,3] n[,3] t[,1] t[,2] t[,3] na[]

11 78 12 85 29 170 2 3 4 3 # Mothersill 1998

75 731 363 714 NA 1 1 3 NA 2 # Reid 1974

2 106 9 205 NA 1 1 3 NA 2 # Slama 1990

58 549 237 1561 NA 1 1 3 NA 2 # Jamrozik 1984

0 33 9 48 NA 1 1 3 NA 2 # Rabkin 1984

3 100 31 98 NA 1 1 3 NA 2 # Richmond 1986

1 31 26 95 NA 1 1 3 NA 2 # Leung 1991

6 39 17 77 NA 1 1 3 NA 2 # Langford 1983

79 702 77 694 NA 1 1 2 NA 2 # Gritz 1992

18 671 21 535 NA 1 1 2 NA 2 # Campbell 1986

64 642 107 761 NA 1 1 3 NA 2 # Sanders 1989

5 62 8 90 NA 1 1 3 NA 2 # Page 1986

20 234 34 237 NA 1 1 3 NA 2 # Vetter 1990

8 116 19 149 NA 1 1 2 NA 2 # Pallonen 1994

95 1107 143 1031 NA 1 1 3 NA 2 # Russell 1983

15 187 36 504 NA 1 1 3 NA 2 # Stewart 1982

78 584 73 675 NA 1 1 3 NA 2 # Russell 1979

69 1177 54 888 NA 1 1 3 NA 2 # Kendrick 1995

20 49 16 43 NA 1 2 3 NA 2 # Decker 1989

7 66 32 127 NA 1 2 4 NA 2 # Mogielnicki 1986

12 76 20 74 NA 1 3 4 NA 2 # Hilleman 1993

9 55 3 26 NA 1 3 4 NA 2 # Gillams 1984

END

# Initial values

list(d=c(NA, 0.0E+00, 0.0E+00, 0.0E+00), mu=c(0.0E+00, 0.0E+00, 0.0E+00, 0.0E+00, 0.0E+00, 0.0E+00, 0.0E+00, 0.0E+00, 0.0E+00, 0.0E+00, 0.0E+00, 0.0E+00, 0.0E+00, 0.0E+00, 0.0E+00, 0.0E+00, 0.0E+00, 0.0E+00, 0.0E+00, 0.0E+00, 0.0E+00, 0.0E+00))

list(d=c(NA, -1.0E+00, 1.0E+00, -1.0E+00), mu=c(-3.0E+00, -3.0E+00, -3.0E+00, -3.0E+00, -3.0E+00, -3.0E+00, -3.0E+00, -3.0E+00, -3.0E+00, -3.0E+00, -3.0E+00, -3.0E+00, -3.0E+00, -3.0E+00, -3.0E+00, -3.0E+00, -3.0E+00, -3.0E+00, -3.0E+00, -3.0E+00, -3.0E+00, -3.0E+00))

list(d=c(NA, 2.0E+00, 2.0E+00, 2.0E+00), mu=c(-3.0E+00, 5.0E+00, -1.0E+00, -3.0E+00, 7.0E+00, -3.0E+00, -4.0E+00, -3.0E+00, -3.0E+00, 0.0E+00, -3.0E+00, -3.0E+00, 0.0E+00, 3.0E+00, 5.0E+00, -3.0E+00, -3.0E+00, -1.0E+00, -3.0E+00, -7.0E+00, -3.0E+00, -3.0E+00))

**Contrast-RE: Contrast-based parameterisation – Random Effects**

# Binomial likelihood, logit link

# Random effects model for multi-arm trials

model{ # *** PROGRAM STARTS

for(i in 1:ns){ # LOOP THROUGH STUDIES

w[i,1] <- 0 # adjustment for multi-arm trials is zero for control arm

delta[i,1] <- 0 # treatment effect is zero for control arm

mu[i] ~ dnorm(0,.0001) # vague priors for all trial baselines

for (k in 1:na[i]) { # LOOP THROUGH ARMS

r[i,k] ~ dbin(p[i,k],n[i,k]) # binomial likelihood

logit(p[i,k]) <- mu[i] + delta[i,k] # model for linear predictor

rhat[i,k] <- p[i,k] * n[i,k] # expected value of the numerators

#Deviance contribution

dev[i,k] <- 2 * (r[i,k] * (log(r[i,k])-log(rhat[i,k]))

+ (n[i,k]-r[i,k]) * (log(n[i,k]-r[i,k]) - log(n[i,k]-rhat[i,k]))) }

# summed residual deviance contribution for this trial

resdev[i] <- sum(dev[i,1:na[i]])

for (k in 2:na[i]) { # LOOP THROUGH ARMS

# trial-specific LOR distributions

delta[i,k] ~ dnorm(md[i,k],taud[i,k])

# mean of LOR distributions (with multi-arm trial correction)

md[i,k] <- d[t[i,k]] - d[t[i,1]] + sw[i,k]

# precision of LOR distributions (with multi-arm trial correction)

taud[i,k] <- tau *2*(k-1)/k

# adjustment for multi-arm RCTs

w[i,k] <- (delta[i,k] - d[t[i,k]] + d[t[i,1]])

# cumulative adjustment for multi-arm trials

sw[i,k] <- sum(w[i,1:k-1])/(k-1)

}

}

totresdev <- sum(resdev[]) # Total Residual Deviance

d[1]<-0 # treatment effect is zero for reference treatment

# vague priors for treatment effects

for (k in 2:nt){ d[k] ~ dnorm(0,.0001) }

sd ~ dunif(0,5) # vague prior for between-trial SD

tau <- pow(sd,-2) # between-trial precision = (1/between-trial variance)

} # *** PROGRAM ENDS

Data as per Contrast-FE.

# Initial values

list(d=c(NA, 0.0E+00, 0.0E+00, 0.0E+00), sd=1.0E+00, mu=c(0.0E+00, 0.0E+00, 0.0E+00, 0.0E+00, 0.0E+00, 0.0E+00, 0.0E+00, 0.0E+00, 0.0E+00, 0.0E+00, 0.0E+00, 0.0E+00, 0.0E+00, 0.0E+00, 0.0E+00, 0.0E+00, 0.0E+00, 0.0E+00, 0.0E+00, 0.0E+00, 0.0E+00, 0.0E+00))

list(d=c(NA, -1.0E+00, 1.0E+00, -1.0E+00), sd=4.0E+00, mu=c(-3.0E+00, -3.0E+00, -3.0E+00, -3.0E+00, -3.0E+00, -3.0E+00, -3.0E+00, -3.0E+00, -3.0E+00, -3.0E+00, -3.0E+00, -3.0E+00, -3.0E+00, -3.0E+00, -3.0E+00, -3.0E+00, -3.0E+00, -3.0E+00, -3.0E+00, -3.0E+00, -3.0E+00, -3.0E+00))

list(d=c(NA, 2.0E+00, 2.0E+00, 2.0E+00), sd=2.0E+00, mu=c(-3.0E+00, 5.0E+00, -1.0E+00, -3.0E+00, 7.0E+00, -3.0E+00, -4.0E+00, -3.0E+00, -3.0E+00, 0.0E+00, -3.0E+00, -3.0E+00, 0.0E+00, 3.0E+00, 5.0E+00, -3.0E+00, -3.0E+00, -1.0E+00, -3.0E+00, -7.0E+00, -3.0E+00, -3.0E+00))

**Arm-FE: Arm-based parameterisation - fixed effects**

model{

#prior on treatment effect betas

beta[1]<-0

#prior on tx effect mean

for (qq in 2:nTx){

beta[qq]~dnorm(0,1.0E-4)

}

# prior on intercept

for(ss in 1:nStudies){

alpha[ss] ~ dnorm(0,1.0E-4)

}

#fit data

for(ii in 1:nObs ){

x[ii] <- alpha[study[ii]] + beta[tx[ii]]

#logit link for probability of response in control and treatment arms

logit(prob[ii]) <- x[ii]

#binomial likelihood

r[ii] ~ dbin(prob[ii], n[ii])

}

}

# Data

list(nTx=4, nStudies=22, nObs=45)

study[] tx[] r[] n[]

1 2 11 78 #Mothersill 1988

1 3 12 85 #Mothersill 1988

1 4 29 170 #Mothersill 1988

2 1 75 731 #Reid 1974

2 3 363 714 #Reid 1974

3 1 2 106 #Slama 1990

3 3 9 205 #Slama 1990

4 1 58 549 #Jamrozik 1984

4 3 237 1561 #Jamrozik 1984

5 1 0 33 #Rabkin 1984

5 3 9 48 #Rabkin 1984

6 1 3 100 #Richmond 1986

6 3 31 98 #Richmond 1986

7 1 1 31 #Leung 1991

7 3 26 95 #Leung 1991

8 1 6 39 #Langford 1983

8 3 17 77 #Langford 1983

9 1 79 702 #Gritz 1992

9 2 77 694 #Gritz 1992

10 1 18 671 #Campbell 1986

10 2 21 535 #Campbell 1986

11 1 64 642 #Sanders 1989

11 3 107 761 #Sanders 1989

12 1 5 62 #Page 1986

12 3 8 90 #Page 1986

13 1 20 234 #Vetter 1990

13 3 34 237 #Vetter 1990

14 1 8 116 #Pallonen 1994

14 2 19 149 #Pallonen 1994

15 1 95 1107 #Russell 1983

15 3 143 1031 #Russell 1983

16 1 15 187 #Stewart 1982

16 3 36 504 #Stewart 1982

17 1 78 584 #Russell 1979

17 3 73 675 #Russell 1979

18 1 69 1177 #Kendrick 1995

18 3 54 888 #Kendrick 1995

19 2 20 49 #Decker 1989

19 3 16 43 #Decker 1989

20 2 7 66 #Mogielnicki 1986

20 4 32 127 #Mogielnicki 1986

21 3 12 76 #Hilleman 1993

21 4 20 74 #Hilleman 1993

22 3 9 55 #Gillams 1984

22 4 3 26 #Gillams 1984

END

Initial values as per Contrast-FE.

**Arm-RE: Arm-based parameterisation – random effects with constant variance**

model{

#prior on random tx effect variance

areSD~dunif(0,5)

reTau <- 2/pow(areSD,2)

#prior on treatment effect betas

beta[1]<-0

#prior on tx effect mean

for (qq in 2:nTx){

beta[qq]~dnorm(0,1.0E-4)

}

#prior on intercept

for(ss in 1:nStudies){

alpha[ss] ~ dnorm(0,1.0E-4)

for (tt in 1:nTx){

re[ss,tt] ~dnorm(0,reTau)

}

}

#fit data

for(ii in 1:nObs ){

x[ii] <- alpha[study[ii]] + beta[tx[ii]]+re[study[ii],tx[ii]]

#logit link for probability of response in control and treatment arms

logit(prob[ii]) <- x[ii]

#binomial likelihood

r[ii] ~ dbin(prob[ii], n[ii])

}

}

Data as per Arm-FE.

Initial values as per CONTRAST-RE.

**Arm-RE Tx. Arm-based parameterisation – random effects with non-constant variance**

model{

#prior on random tx effect variance

#prior on treatment effect betas

beta[1]<-0

areSD[1]~dunif(0,5)

reTau[1] <- 2/pow(areSD[1],2)

#prior on tx effect mean and random tx effect variance

for (qq in 2:nTx){

beta[qq]~dnorm(0,1.0E-4)

areSD[qq]~dunif(0,5)

reTau[qq] <- 2/pow(areSD[qq],2)

}

#prior on intercept

for(ss in 1:nStudies){

alpha[ss] ~ dnorm(0,1.0E-4)

for (tt in 1:nTx){

re[ss,tt] ~dnorm(0,reTau[tt])

}

}

#fit data

for(ii in 1:nObs ){

x[ii] <- alpha[study[ii]] + beta[tx[ii]]+re[study[ii],tx[ii]]

#logit link for probability of response in control and treatment arms

logit(prob[ii]) <- x[ii]

#binomial likelihood

r[ii] ~ dbin(prob[ii], n[ii])

}

}

Data as per Arm-FE.

# Initial values

list(beta=c( NA, 0.00000E+00, 0.00000E+00, 0.00000E+00), areSD=c(1.00000E+00, 1.00000E+00, 1.00000E+00, 1.00000E+00), alpha=c(0.00000E+00, 0.00000E+00, 0.00000E+00, 0.00000E+00, 0.00000E+00, 0.00000E+00, 0.00000E+00, 0.00000E+00, 0.00000E+00, 0.00000E+00, 0.00000E+00, 0.00000E+00, 0.00000E+00, 0.00000E+00, 0.00000E+00, 0.00000E+00, 0.00000E+00, 0.00000E+00, 0.00000E+00, 0.00000E+00, 0.00000E+00, 0.00000E+00))

list(beta=c( NA, -1.00000E+00, 1.00000E+00, -1.00000E+00), areSD=c(4.00000E+00, 4.00000E+00, 4.00000E+00, 4.00000E+00), alpha=c(-3.00000E+00, -3.00000E+00, -3.00000E+00, -3.00000E+00, -3.00000E+00, -3.00000E+00, -3.00000E+00, -3.00000E+00, -3.00000E+00, -3.00000E+00, -3.00000E+00, -3.00000E+00, -3.00000E+00, -3.00000E+00, -3.00000E+00, -3.00000E+00, -3.00000E+00, -3.00000E+00, -3.00000E+00, -3.00000E+00, -3.00000E+00, -3.00000E+00))

list(beta=c( NA, 2.00000E+00, 2.00000E+00, 2.00000E+00), areSD=c(2.00000E+00, 2.00000E+00, 2.00000E+00, 2.00000E+00), alpha=c(-3.00000E+00, 5.00000E+00, -1.00000E+00, -3.00000E+00, 7.00000E+00, -3.00000E+00, -4.00000E+00, -3.00000E+00, -3.00000E+00, 0.00000E+00, -3.00000E+00, -3.00000E+00, 0.00000E+00, 3.00000E+00, 5.00000E+00, -3.00000E+00, -3.00000E+00, -1.00000E+00, -3.00000E+00, -7.00000E+00, -3.00000E+00, -3.00000E+00))

***Model extensions***

The following section illustrates how commonly employed model extensions can be incorporated in to the arm-based parameterization.

*Alternative data and treatment effect scales*

In the main text we consider the analysis of binary data with treatment effects constrained to be consistent on the log odds scale. Other data types can be incorporated by substituting appropriate distributions for equation 1. Treatment effects can be constrained to be consistent on a variety of scales by substituting alternative ‘inverse link’ functions for equation 2. For example, treatment effects for continuous outcomes could be constrained to be consistent on a linear scale by substituting a normal distribution for equation 1 and an identity link for equation 2.

A table of some common distributions and the inverse link functions corresponding to various treatment effect scales are given in the next section.

*Data reported as contrast statistics*

In some cases, treatment response data may be reported as the difference between two treatment arms (contrast-level statistics) rather than the response observed within an individual treatment arm (arm-level statistics).

Contrast statistics can be modelled using the arm-based parameterisation by converting contrast-level statistics to arm-level statistics. The mean responses for individual study arms are calculated by setting the response for the base treatment arm from each trial to zero. The contrast statistics comparing the remaining treatments to this base treatment can then be interpreted as absolute responses for these treatments.

Deriving the variances for these arm-level statistics is more involved. The variance of a contrast statistic $x_{s}$ from study *s* (${se}_{x_{s}}^{2})$ is the sum of the variances for the responses in the individual arms. For a trial comparing treatments A and B and reporting a standard error for the contrast statistic of ${se}_{a,b}$, standard errors for the mean response for each trial armcan be estimated as:

$${se}_{a}={se}_{b}=\sqrt{\left( \left( {se}_{a,b}^{2} \right)/2 \right)}$$

For trials with three or more arms, standard errors for the mean response for each trial arm can be estimated by solving a set of simultaneous equations. For example, if we consider a three-arm trial comparing treatments A, B, and C the standard errors for each individual arm would be:

$${se}_{a}=\sqrt{\left( \left( {se}_{a,b}^{2}+{se}_{a,c}^{2}-{se}_{b,c}^{2} \right)/2 \right)}$$

$${se}_{b}=\sqrt{{se}_{a,b}^{2}-{se}_{a}^{2}}$$

$${se}_{c}=\sqrt{{se}_{a,c}^{2}-{se}_{a}^{2}}$$

For a worked example see Woods et al., (2010). If standard errors are not available for all contrasts (for example contrast statistics are reported for arm B vs. A and C vs. A only) then an approximation is required to derive arm-level standard errors. For a suggested approximation see Woods et al., (2010). In some instances multiple contrast statistics may have been obtained from a single regression analysis. If the variance-covariance matrix for the regression analysis is available then the variance for arm A can be estimated as the covariance between the contrast comparing arm A to B and the contrast comparing arm A to C. The variances for arms B and C can then be estimated using the equations above.

The resulting arm-level data can then be analysed using the methods presented in this paper, however the $\mu_{s[i]}$ can no longer be interpreted as providing information on the base or reference treatment response. For typical contrast-level statistics such as odds ratios or hazard ratios equation 1 is replaced with a normal distribution ($x_{i}\sim N\left( \eta_{i},{se}_{x_{i}}^{2} \right)$) where $x_{i}$ is the log odds/hazard of response in arm *i* and ${se}_{x_{i}}$ its standard error. The link function (equation 2) is also removed as an identity link is appropriate. We note that this approach treats the estimated standard error as the true standard error. Although this is standard practice (Dias et al., 2014), the implications of this assumption for the estimates derived from network meta-analysis are unknown and require further research.

Note that this approachnsures that correlations in the underlying contrast data are preserved. In a network meta-analysis using the contrast-based parameterization this requires use of a multivariate distribution (even for fixed effects analysis). Current implementations of this approach (Dias et al., 2014) require data from studies with different numbers of arms to be included in separate loops within the analysis code. Separate loops are required to allow the dimensions of the multivariate normal likelihood to vary according to the number of contrast statistics in each study (which will be one less than the number of trial arms in each study) (see Dias et al., 2014 p93). The arm-based parameterisation can be used directly once contrast statistics are converted to arm-specific statistics (Woods et al., 2010). This has the benefit of not requiring any further complication of the analysis code.

*Analysis of consistency*

When conducting network meta-analysis we should expect and expose the effects of heterogeneity by looking for empirical evidence of inconsistency between treatment effects estimated using direct and indirect data.

A number of models have been put forward to examine inconsistency. For example, in Dias et al. (2013a), independent treatment effects are estimated for each treatment comparison for which trial evidence is available. Where trials have more than two treatment arms, independent treatment effects are estimated for the comparisons against a common reference arm rather than for all the possible comparisons. This is necessary because by definition the treatment effect estimates are consistent within a trial. The DIC and other measures of fit can be compared between this inconsistency model and the network meta-analysis (consistency) model to ascertain whether relaxing the consistency constraint has significantly improved the model fit.

To implement this inconsistency model in the arm-based parameterisation equation 7 is replaced by:

$\eta_{i}=\text{µ}_{s\left[i\right]}+d_{t\left[i\right],b\left[i\right]}$ if t[i] ≠b[i], otherwise

$$\eta_{i}=\text{µ}_{s\left[i\right]}$$

Treatment numbers are assigned to each treatment. The base treatment in each trial *b[i]* is (arbitrarily) the lowest treatment index for study *s[i].*

An alternative model is the ‘design by treatment interaction’ model (Higgins et al., 2012). ‘Design’ refers to the combination of treatments included within a study. The different study designs (combinations of treatments) included within the analysis are indexed by *z[i]=1,2,…,Z* where Z is the number of unique study designs. An additional parameter *ω_z[i], t[i ]_* is added to the network meta-analysis model to account for the deviation between the estimated treatment effects that are constrained to be consistent across the network and those that are specific to the particular study designs. The model in equation 7 is replaced with:

$$\eta_{i}=\text{µ}_{s[i]}+d_{t[i]}+\omega_{z[i],t[i]}$$

$\omega_{z[i],t[i]}$is constrained to zero for the lowest indexed treatment within a design to ensure the analysis is identified within a design. $\omega_{z[i],t[i]}$, is only estimable where a treatment comparison is duplicated. Taking our example analysis and working from top to bottom of Figure 1, we can see that there are five designs which are unique (i.e. do not contain the same comparator set as any other trial). These correspond to the Cottraux et al. (1983), Williams & Hall (1988), Decker & Evans (1989), Mogielnicki et al. (1986), and Mothersill et al. (1988).

Again, measures of model fit can be compared between the full design-by-treatment interaction model (the model incorporating the ($\omega_{z[i],t[i]}$)) and the network meta-analysis model to ascertain whether relaxing the consistency constraint improves model fit.

A further method for assessing inconsistency is the node-splitting approach which involves estimating treatment effects based on the direct and indirect evidence separately for each comparison where such evidence exists (Dias et al., 2010). This can be implemented in the arm-based parameterization by amending the treatment indexes for the treatments of interest in trials where they are directly compared. They should be given unique indexes so that independent direct and indirect estimates can be obtained. The program code does not need to be changed.

**Meta-regression**

As well as exposing the effects of heterogeneity we should try to explain and account for observed heterogeneity. Meta-regression allows us to do this by including study-level covariable terms as treatment effect interactions (Dias et al., 2013b).

Meta-regression can be implemented with independent treatment-specific covariable interaction effects (*β_t_*). Equation 7 is replaced with:

$\eta_{i}=\text{µ}_{s\left[i\right]}+d_{t\left[i\right].\overline{x}}+\beta_{t[i]}\left(x_{s\left[i\right]}-\overline{x}\right)$ and $\beta_{1}=0$

$x_{s\left[i\right]}$is the value of the covariable for study s[i] and $\overline{x}$is the mean value of this variable across studies. The treatment effect is now the effect at the average value of the covariable ($d_{t\left[i\right].\overline{x}}$). This approach allows covariable effects to vary across treatments and will produce the same inferences regardless of the choice of reference treatment. Again the notation and coding is simpler with the arm-based parameterization. This model requires that there are sufficient studies available for each comparison to allow all interaction effectsto be estimated.

Alternatively, a common covariable effect ($\beta$) can be applied across treatments:

and $\beta_{1}=0$

The results now depend on the choice of reference treatment as only trials that include the reference treatment contribute to the estimation of the interaction effect (the model is not parameterization invariant).

The model can also be implemented assuming interaction effects are exchangeable with a random effect variance ($\sigma_{B}^{2}$):

$\beta_{2..T}\sim N(\beta,\sigma_{B}^{2})$ and $\beta_{1}=0$.

***Alternative likelihoods and scales for synthesis***

Table 3a: Alternative likelihood functions and treatment effect scales

| **Likelihood** | **Scale** | **Link function** |
| --- | --- | --- |
| Binomial | Arcsine | $p_{i}=\sin(\eta_{i})^{2}$ |
|  | Hazard ratio event | $p_{i}=\exp(-\exp(\eta_{i}))$ |
|  | Hazard ratio no event | $p_{i}=1-\exp(-\exp(\eta_{i}))$ |
|  | Probit | $p_{i}=\Phi(\eta_{i})$ |
|  | Relative risk event | $p_{i}=\exp(\eta_{i})$ |
|  | Relative risk no event | $p_{i}=1-\exp(\eta_{i})$ |
|  | Risk difference | $p_{i}=\eta_{i}$ |
|  | Odds ratio | $p_{i}=\frac{\exp(\eta_{i})}{1+\exp(\eta_{i})}$ |
| Poisson $r_{i}\sim poisson\left(\lambda_{i}E_{i}\right)$ | Rate ratio | $\lambda_{i}=\exp(\eta_{i})$ |
| Normal $y_{i}\sim N\left(\overline{y_{i}},se_{i}^{2}\right)$ | Absolute difference | $y_{i}=\eta_{i}$ |
|  | Ratio of means | $y_{i}=\exp(\eta_{i})$ |

For a binary variable *r_i_*, *n_i_* and *p_i_* are the number of events, number of patients and probability of an event for arm *i*. For a count variable *r_i_*, *λ_i_* and *E_i_* are the number of events, rate at which events occur and exposure time across all individuals for arm i. For a continuous outcome $\bar{y}_{i}$, ${se}_{i}^{2}$and *y_i_* are the mean outcome observed, observed variance and predicted outcome for arm *i*.

**Additional references not included in main manuscript**

Higgins, J., Jackson, D., Barrett, J., Lu, G., Ades, A., White, I. (2012) Consistency and inconsistency in network meta-analysis: concepts and models for multi-arm studies. *Res Synth Methods*. 3(2). p98-110.

**References for smoking cessation studies**

Campbell, I.A., Hansford, M., Prescott, R.J. (1986) Effect of a "stop smoking" booklet on smokers attending for chest radiography a controlled study. *Thorax*. 41(5). p369-71.

Cottraux, J.A., Harf, R., Boissel, J.P., Schbath, J., Bouvard, M., Gillet, J. (1983) Smoking cessation with behaviour therapy or acupuncture; a controlled study. *Behav Res Ther.* 21(4). p417-424.

Decker, B.D., Evans, R.G. (1989) Efficacy of a minimal contact version of a multimodal smoking cessation program. *Addict Behav*. 14(5). p487-91.

Gillams, J., Lewith, G.T., Machin, D. (1984) Acupuncture and group therapy in stopping smoking. *Practitioner*. 228(1389). p341-4.

Gritz, E.R., Berman, B.A., Bastani, R., Wu, M. (1992) A randomized trial of a self-help smoking cessation intervention in a nonvolunteer female population: testing the limits of the public health model. *Health Psychol*. 11(5). p280-9.

Hilleman, D.E., Mohiuddin, S.M., Delcore, M.G., Lucas, B.D. Jr.(1993) Randomized, controlled trial of transdermal clonidine for smoking cessation. *Ann Pharmacother*. 27(9). p1025-8.

Jamrozik, K., Vessey, M., Fowler, G., Wald, N., Parker, G., Van Vunakis, H. (1984) Controlled trial of three different antismoking interventions in general practice. *Br Med J*. 288(6429). p1499-1503.

Kendrick, J.S., Zahniser, S.C., Miller, N., et al.(1995) Integrating smoking cessation into routine public prenatal care results of the smoking cessation in pregnancy project. *Am J Public Health*. 85(2). p217-22.

Langford, E.R., Thompson, E.G., Tripp, S.C. (1983) Smoking and health education during pregnancy: evaluation of a program for women in prenatal classes. *Can J Public Health.* 74(4). p285- 9.

Leung JP.(1991) Smoking cessation by auricular acupuncture and behavioral therapy. *Psychologia Int J Psychol Orien.* 34. p177-87

Mogielnicki, R.P., Neslin, S., Dulac, J., Balestra, D., Gillie, E., Corson, J. (1986) Tailored media can enhance the success of smoking cessation clinics. J Behav Med. 9(2). p141-61.

Mothersill, K.J., McDowell, I., Rosser, W. (1988) Subject characteristics and long term post-program smoking cessation. *Addict Behav*. 13(1). p29-36.

Page, A.R., Walters, D.J., Schlegel, R.P., Best, J.A. (1986) Smoking cessation in family practice the effects of advice and nicotine chewing gum prescription. *Addict Behav*. 11(4). p443-6.

Pallonen, U.E., Leskinen, L., Prochaska, J.O., Willey, C.J., Kaari-ainen, R., Salonen, J.T.(1994) A 2-year self-help smoking cessation manual intervention among middle-aged Finnish men: an application of the transtheoretical model. *Prev Med*. 23(4). p507-14.

Rabkin SW, Boyko E, Shane F, Kaufert J A.(1984) randomized trial comparing smoking cessation programs utilizing behavior modifications, health education or hypnosis. *Addict Behav*. 9(2): 157-73.

Reid, D.D., Brett, G.Z., Hamilton, P.J.S., Jarrett, R.J., Keen, H., Rose, G. (1974) Cardiorespiratory disease and diabetes among middle-aged male civil servants. *Lancet.* 303(7856). p469-73.

Richmond, R.L., Austin, A., Webster, I.W. (1986) Three year evaluation of a programme by general practitioners to help patients to stop smoking. *Br Med J*. 292(6523). p803-6

Russell, M.A., Merriman, R., Stapleton, J., Taylor, W. (1983) Effect of nicotine chewing gum as an adjunct to general practitioners’ advice against smoking. *Br Med J*. 287(6407). p1782-5.

Russell, M.A., Wilson, C., Taylor, C., Baker, C.D. (1979) Effect of general practitioners’ advice against smoking. *Br Med J*. 2(6184). p231- 5.

Sanders, D., Fowler, G., Mant, D., Fuller, A., Jones, L., Marzillier, J. (1989) Randomized controlled trial of anti-smoking advice by nurses in general practice*. JR Coll Gen Pract*. 39(324). p273- 6.

Slama, K., Redman, S., Perkins, J., Reid, A.L., Sanson-Fisher, R.W. (1990) The effectiveness of two smoking cessation programmes for use in general practice a randomised clinical trial. *Br Med J*. 300(6741). p1707-9.

Stewart, P.J., Rosser, W.W. (1982) The impact of routine advice on smoking cessation from family physicians. *Can Med Assoc J*. 126(9). p1051-4.

Vetter, N.J., Ford, D.(1990) Smoking prevention among people aged 60 and over a randomized controlled trial. *Age Ageing*. 19(3). p164-8.

Williams, J.M., Hall, D.W. (1988) Use of single session hypnosis for smoking cessation. *Addict Behav*. 13(2). p205-8.
